# Urban-rural differences in the successful aging among older adults in China

**DOI:** 10.1371/journal.pone.0319105

**Published:** 2025-03-20

**Authors:** Keying Song, Jiedan Luo, Xiaodong Cao, Zijian Zhao

**Affiliations:** 1 Gdansk University of Physical Education and Sport, Gdansk, Poland; 2 Zhengzhou University, Zhengzhou, China; Universidad Nacional Autonoma de Mexico, MEXICO

## Abstract

This study aimed to reveal urban-rural disparities in successful aging among Chinese older adults and the impact of gender and age on aging outcomes. We utilized the Successful Aging Index (SAI), a multidimensional measure encompassing social, economic, bio-clinical, psychological, and lifestyle factors. Scores on the SAI range from 0 to 10, with higher scores signifying better aging. Data was sourced from the 2018 Chinese Longitudinal Healthy Longevity Survey, comprising 7,315 participants. Urban older adults (OU) had significantly higher SAI scores than rural older adults (OR), with averages of 4.32 ±  1.44 and 3.85 ±  1.24, respectively (p < 0.001). Men showed more successful aging than women, regardless of their residence (p < 0.001). OU had better financial and educational status and higher social activity scores, except for friend interaction (p < 0.001). They were more physically active (p < 0.001), more adherent to the Mediterranean diet (p < 0.001), and less likely to smoke (p = 0.018). However, OU had a higher prevalence of cardiovascular disease risk factors compared to OR (p < 0.001). Notably, depression scores were similar between OU and OR (p = 0.129). In summary, significant urban-rural differences in successful aging are evident among Chinese older adults, with urban-dwelling older adults aging more successfully than their rural peers. Men, irrespective of their place of residence, experience more successful aging outcomes than women.

## Introduction

The aging of the global population is the most important medical and social demographic problem worldwide [1]. Since the end of the 20th century, China has indeed entered an aging society, with older adults population continuously increasing. According to the seventh population census conducted in 2020, individuals aged 65 and above accounted for 13.50% of the total population, which is approximately 190 million people. This represents a significant increase of 4.63 percentage points compared to the 2010 census and an even more substantial increase of 6.54 percentage points compared to the 2000 census [2,3]. The significant rise in both the proportion and total number of older individuals presents a growing challenge to the healthcare system. Consequently, the issue of successful aging, along with the urban-rural disparities in this process, has become increasingly significant as urbanization accelerates. It is therefore both urgent and critical to assess the levels of successful aging among older adults in both urban and rural China, to develop strategies that foster successful aging, and to enhance the overall quality of life for this demographic.

As of now, there is no universally recognized definition of successful aging [4,5]. Successful aging was originally defined as the low risk and good physiological function who exhibit little or no age and disease related attenuation [6] and successful aging should include the following three elements: avoiding disease and disability; maintain good physiological and psychological function and sustained social participation [7]. The World Health Organization (WHO) has defined healthy aging as a process of maintaining functional ability to enable well-being in older age. Recently, the definition has been altered to better describe its multi-factorial nature, suggesting that successful aging not only depends on objective and biological factors such as disease, disability, and cognitive function, but also subjective and psychological, social factors such as subjective well-being, social participation, psychological resources, and self-assessment of successful aging [8]. A large number of studies have demonstrated that, a broad range of factors such as education [9], activity/exercise [10,11], sustained engagement in social and productive activities [7,12], physical [11,13], psychological [11,14], emotional as well as psychosocial functioning [15] and dietary patterns [11,16] are associated with or leading to successful or healthy aging while barriers such as social isolation, perceived reduced status and false stereotype of health prevents one’s capacity to age successfully [17]. For example, M. M. Dahany’s study showed that physical and psychological well-being, regular exercise and nutritional status are major determinants of successful aging in French population [11]. Tyrovolas S’s study also confirmed the multiple dimensions of aging by applying factor analysis on the components of the index extracted three main components for successful aging: psychosocial-economic, bioclinical and lifestyle [5]. All these have established the theoretical foundation for our study. In summary, we consider successful aging to be a comprehensive concept that not only focuses on the physical health of older adults but also includes their psychological well-being, social participation, lifestyle and quality of life.

In the present work, we followed the multi-dimensional approach of successful aging reported by experts [18–20], and created the successful aging index (SAI), which includes 10 components covering social and economical characteristics (i.e., education, financial status, participation in social activities with friends, with others and yearly excursions), bio-clinical characteristics (BMI, CVD risk factors), psychological characteristics (GDS score) and lifestyle characteristics (physical activity and Med Diet Score). The SAI has been developed and validated by Stefanos Tyrovolas [5]. Finally, we made a comparison between successful aging in urban/rural settings and between socio-demographic variables: gender and age.

## Materials and methods

### Sample

Our dataset originates from the seventh wave of the Chinese Longitudinal Healthy Longevity Survey (CLHLS) conducted in 2018, covering 23 of China’s 31 provinces. The survey included 15,874 respondents over 65 years old and comprised over 750 questions. The CLHLS was approved by the Ethics Committee of Peking University (IRB00001052-13074), and all procedures adhered to the ethical standards of the committee and the 1964 Helsinki declaration. More detailed information about the CLHLS and the details of the sampling design, response rates, attrition, and systematic assessments of data quality were available elsewhere [21].

For our analysis, we selected approximately 50 questions to construct our indexes. Samples were excluded if any question was left unanswered or marked as unknown, or if they contained obvious errors. Finally, our sample consisted of 7,315 men and women from urban and rural China, with 4,321 urban dwellers and 2,994 rural residents. All participants provided informed consent for the publication of their information in an online open-access format.

### Measurements

#### Social and economical characteristics.

In the present study, social and economic characteristics encompassed age (measured in years), gender (male/female), place of residence (urban/rural), education (years of schooling), and financial status (self-reported). Participants were asked to rate their economic status relative to other local residents using a five-point scale, from “very rich” to “very poor”. Respondents who reported being “very rich” or “rich” were considered to have a satisfactory financial status.

Furthermore, to assess participants’ social engagement, we recorded the frequency of their social activities with friends and their participation in organized social activities on a scale of “almost every day” (1), “not daily but at least once a week” (2), “not weekly but at least once a month” (3), “not monthly but sometimes” (4), to “never” (5). Additionally, the number of excursions taken in the past two years was documented.

#### Bio-clinical characteristics.

Weight and height were measured using standard procedures to calculate the Body Mass Index (BMI) in kilograms per square meter (kg/m^2^). Obesity was defined as a BMI greater than 29.9 kg/m^2^. Participants self-reported whether they suffered from hypertension, diabetes, and dyslipidemia. We developed a cumulative variable ranging from 0 to 4 to indicate the overall burden of classical cardiometabolic risk factors, including obesity, hypertension, diabetes, and dyslipidemia. Participants received a score of 0 if they had none of the aforementioned risk factors, 1 if they had one factor, and so on.

#### Psychological assessment.

Psychological assessments were conducted using the self-report Geriatric Depression Scale (GDS), which ranges from 0 to 10. Participants responded to ten questions related to depression over the past one to two weeks, using a five-point scale where “always/very good” was scored as 1, “often/good” as 2, “sometimes/so so” as 3, “seldom/bad” as 4, and “never/very bad” as 5. For the calculation of GDS scores, responses were coded with values of 0, 0.25, 0.5, 0.75, and 1 corresponding to the scale points from 1 to 5. Lower GDS scores indicate more severe depressive symptoms.

#### Lifestyle assessment.

The assessment of lifestyle encompassed an evaluation of dietary habits and physical activity. Dietary habits were evaluated using a semi-quantitative, validated, and reproducible food frequency questionnaire (FFQ) [22]. Participants reported their consumption frequency of various food groups—including meat, eggs, fish, milk and dairy products, fruits, vegetables, nuts, beans, staple foods, oils, mushrooms, and algae—on a daily, weekly, or monthly basis. To determine adherence to the Mediterranean diet, a developed and validated Mediterranean Diet Score (Med Diet Score) was utilized, with a theoretical range of 0–44, where higher scores signify greater adherence [23]. Physical activity levels were self-reported, with individuals who responded “yes” to current exercise routines classified as “physically active,” and those who did not as “physically inactive.”

#### Successful aging index.

Successful aging is a multifaceted concept, encompassing biomedical, social, psychological, and subjective aspects. We utilized a comprehensive, previously validated Successful Aging Index (SAI) to measure this phenomenon [5]. The SAI includes socioeconomic status, psychological well-being, lifestyle factors, and bio-clinical indicators such as education, finances, social engagement, physical activity, adherence to the Mediterranean diet, BMI, depression, and cardiovascular risk factors. Each of the 10 components was rated on a scale from 0 to 1, with scores reflecting their impact on successful aging, as informed by current literature [5]. The overall SAI is the sum of these individual scores, with higher totals indicating greater success in aging [5].

The description and coding of SAI are shown as Table 1.

**Table 1:** Description and coding of SAI (total theoretical score 0-10).

Characteristics	Cut points and scores
Social and economical	
Education (years of school)	0-2 years = 0, 3-6 years = 0.33, 7-12 years = 0.66, ≥ 13 years = 1
Satisfactory financial status	Poor/very poor = 0,so so = 0.33,rich = 0.66,very rich = 1
How often series, interact with friends	never = 0, not monthly but sometimes = 0.25, not weekly but at least once for a month = 0.5, not daily but once for a week = 0.75, almost everyday = 1
How often take part in some social activities	never = 0, not monthly but sometimes = 0.25, not weekly but at least once for a month = 0.5, not daily but once for a week = 0.75, almost everyday = 1
Times of excursions in the past two years	0 time = 0, 1-2 times = 0.25, 3-4 times = 0.5, 5-10 times = 0.75, ≥ 11 times = 1
Bio-clinical	
BMI (Kg/m^2^)	normal weight = 1, overweight = 0.5, underweight or obese = 0
CVD risk factors (0-4)	None = 1, 1 factor = 0.75, 2 factors = 0.5, 3 factors = 0.25, 4 factors = 0
Psychological	
GDS score (0-10)	0-5.75 points = 0, 6-6.75 points = 0.25, 7-7.5 points = 0.50, 7.75-8.25 points = 0.75, 8.5-10 points = 1
Lifestyle	
Med Diet Score (0-44)	0-22 points = 0, 23-26 points = 0.25, 27-29 points = 0.50, 30-33 points = 0.75, 34-44 points = 1
Physical activity	Yes = 1, No = 0

### Statistical analysis

Continuous variables are presented as mean±standard deviation (SD) and categorical variables as frequencies. Comparisons between categorical variables were tested using the chi-square test, while comparisons of continuous variables between groups were performed using the independent samples t test for normally distributed variables and the Mann-Whitney U test for non-normally distributed variables. Scatter plots with quadratic lines were used to graphically represent the relationship between SAI index (y-axis) and age (x-axis) by place of residence (urban or rural). SPSS software version 19 (SPSS Inc.,Chicago,IL,USA) was used for all calculations.

## Results

Table 2 presents social-economical, bio-clinical, psychological, and lifestyle characteristics of the study participants, i.e., the older adults living in urban areas (OU) and older adults living in rural areas (OR).

**Table 2:** Characteristics of n = 7,315 participants, based on their place of residence.

	All (n = 7,315)	Urban (n = 4,321)	Rural (n = 2,994)	Effect size	p
Social and economical					
Age (years)	82.8 ± 11.3	83.1 ± 11.3	82.3 ± 11.4	0.07	0.007
Men (%)	47.5	48.9	45.5	1.07	0.004
Education (years)	3.9 ± 4.5	4.7 ± 4.8	2.8 ± 3.6	0.45	<0.001
Satisfactory financial status (%)	21	23.9	16.8	1.42	<0.001
How often series, interact with friends					<0.001
1-almost everyday (%)	29.1	26.4	32.9	0.80	
2-not daily but once for a week (%)	16.9	16.2	17.9	0.91	
3-not weekly but at least once for a month (%)	7.1	7.2	7.0	1.03	
4-not monthly but sometimes (%)	8.9	9.9	7.4	1.34	
5-never (%)	38	40.3	34.7	1.16	
How often take part in organized social activities					<0.001
1-almost everyday (%)	3.1	4.0	1.7	2.35	
2-not daily but once for a week (%)	3.1	4.0	1.8	2.22	
3-not weekly but at least once for a month (%)	3.6	4.7	2	2.35	
4-not monthly but sometimes (%)	6.6	8.0	4.6	1.74	
5-never (%)	83.6	79.3	89.8	0.88	
Times of excursions in the past two years					<0.001
0 time (%)	84.6	80.9	90.0	0.90	
1 time (%)	5	5.6	4.0	1.40	
2 times (%)	4.4	5.5	2.8	1.96	
3-5 times (%)	4.4	5.8	2.5	2.32	
>5 times (%)	1.6	2.2	0.7	3.14	
Bio-clinical					
Hypertension (%)	43.7	46.3	39.9	1.16	<0.001
Diabetes (%)	11	13.8	6.8	2.03	<0.001
Dyslipidemia (%)	6.5	8.6	3.6	2.39	<0.001
BMI(Kg/m^2^)	22.7 ± 4.4	22.9 ± 4.5	22.5 ± 4.3	0.09	0.001
Obese (%)	3.9	4.1	3.5	1.17	0.182
CVD risk factors (0-4)	0.65 ± 0.77	0.73 ± 0.82	0.54 ± 0.69	0.25	<0.001
Psychological					
GDS score (0-10)	7.03 ± 1.51	7.05 ± 1.53	7.00 ± 1.48	0.03	0.129
Lifestyle					
Physical activity (%)	36.6	41.9	28.9	1.45	<0.001
Current smokers (%)	16.7	15.8	17.9	0.88	0.018
Med Diet Score (0-44)	27.45 ± 6.38	28.64 ± 6.50	25.72 ± 5.77	0.475	<0.001
Successful aging index (0-10)	4.13 ± 1.38	4.32 ± 1.44	3.85 ± 1.24	0.35	<0.001

Values are presented as percent (%) or mean ±  standard deviation.

Effect size is measured by Cohen’s d for t-test and by the relative difference between two ratios for categorical variables.

p: p-values derived from Pearson’s t-test for continuous variables or chi-square test for the categorical variables.

### Social-economical, bio-clinical, psychological, and lifestyle characteristics

Urban-dwelling older adults (OU) were likely to be slightly older, with an average age of 83.1 ±  11.3 years, compared to their rural counterparts (OR) at 82.3 ±  11.4 years (p = 0.007). Financially, OU had a higher status, with 23.9% having a higher financial status compared to 16.8% of OR (p < 0.001). Educational levels were also higher among OU, with an average of 4.7 ±  4.8 years of education, compared to 2.8 ±  3.6 years for OR (p < 0.001).

In terms of social activities, OU were more socially active, particularly in going on excursions and participating in organized social activities, but less so in terms of interacting with friends regularly. Specifically, OU were more likely to have gone on one or more excursions in the past two years (19.1% vs. 10.0% of total participants for each group, p < 0.001) and to participate in organized social activities more frequently (20.7% vs. 10.2% of total participants for each group, p < 0.001). However, they were less likely to interact with friends on at least a monthly basis (49.8% vs. 57.9% of total participants for each group, p < 0.001).

Regarding lifestyle, a higher percentage of OU were physically active compared to OR (41.9% vs. 28.9% of total participants for each group, p < 0.001), and there were fewer current smokers among OU than OR (15.8% vs. 17.9%, p = 0.018). Additionally, OU had a higher adherence to the Mediterranean diet, with an average score of 28.64 ±  6.50 compared to 25.72 ±  5.77 for OR (p < 0.001), a trend that was consistent across both sexes (Table 3).

**Table 3:** Characteristics of n =  7,315 participants in total, based on their place of residence and their gender (men, women).

	Urban (n = 4,321)		Rural (n = 2,994)		p1	p2
	Men (n = 2,113)	Women (n = 2,208)	Men (n = 1,363)	Women (n = 1,631)		
Social and economical						
Age (years)	82.1 ± 10.8	84.0 ± 11.7	81.0 ± 10.6	83.5 ± 11.9	0.002	0.197
Education (years)	6.4 ± 4.8	3.1 ± 4.3	4.2 ± 3.6	1.5 ± 3.1	<0.001	<0.001
Satisfactory financial status (%)	27.3	20.7	18.8	15.2	<0.001	<0.001
How often series with friends					<0.001	<0.001
1-almost everyday (%)	25.1	27.6	33.5	32.4		
2-not daily but once for a week (%)	17.7	14.9	18.5	17.5		
3-not weekly but at least once for a month (%)	7.8	6.6	7.1	6.9		
4-not monthly but sometimes (%)	11.4	8.4	7.5	7.4		
5-never (%)	38.1	42.5	33.4	35.8		
How often take part in organized social activities					<0.001	<0.001
1-almost everyday (%)	4.1	4.0	2.1	1.4		
2-not daily but once for a week (%)	4.3	3.8	1.9	1.8		
3-not weekly but at least once for a month (%)	5.8	3.6	2.8	1.3		
4-not monthly but sometimes (%)	9.6	6.4	5.4	3.9		
5-never (%)	76.2	82.2	87.8	91.5		
Times of excursions past two years					<0.001	<0.001
0 time (%)	78.5	83.1	88.4	91.4		
1 time (%)	5.3	6.0	4.2	3.9		
2 times (%)	7.1	4.0	3.9	1.8		
3-5 times (%)	6.3	5.3	2.8	2.5		
>5 times (%)	2.8	1.6	0.7	0.4		
Bio-clinical						
Hypertension (%)	44.9	47.6	38.2	41.4	<0.001	<0.001
Diabetes (%)	12.9	14.7	6.2	7.4	<0.001	<0.001
Dyslipidemia (%)	7.5	9.6	4.0	3.3	<0.001	<0.001
BMI (Kg/m^2^)	23.0 ± 4.2	22.7 ± 4.8	22.7 ± 3.9	22.4 ± 4.6	0.017	0.018
Obese (%)	3.6	4.6	2.6	4.2	0.119	0.564
CVD risk factors (0-4)	0.69 ± 0.78	0.77 ± 0.85	0.51 ± 0.68	0.56 ± 0.69	<0.001	<0.001
Psychological						
GDS score (0-10)	7.22 ± 1.50	6.89 ± 1.54	7.15 ± 1.42	6.87 ± 1.52	0.195	0.625
Lifestyle						
Physical activity (%)	47.2	36.8	33.2	25.3	<0.001	<0.001
Current smokers (%)	27.8	4.4	33.9	4.6	<0.001	0.761
Med Diet Score (0-44)	29.21 ± 6.24	28.10 ± 6.70	26.29 ± 5.72	25.26 ± 5.77	<0.001	<0.001
Successful aging index (0-10)	4.62 ± 1.41	4.03 ± 1.41	4.13 ± 1.25	3.62 ± 1.18	<0.001	<0.001

Values are presented as percent (%) or mean ±  standard deviation.

P:p-values derived from Pearson’s t-test for continuous variables or chi-square test for the categorical variables.

p1:for the comparisons between men living in rural and men living in urban and.

p2:for the comparisons between women living in rural and women living in urban.

Regarding bio-clinical characteristics, urban-dwelling older adults (OU) exhibited a higher risk of developing cardiometabolic diseases compared to their rural counterparts (OR), with an average of 0.73 ±  0.82 versus 0.54 ±  0.69 in the number of cardiovascular disease (CVD) risk factors (p < 0.001). The prevalence of several conditions was significantly higher among OU: hypertension (46.3% vs. 39.9%, p < 0.001), diabetes (13.8% vs. 6.8%, p < 0.001), dyslipidemia (8.6% vs. 3.6%, p < 0.001), and obesity (4.1% vs. 3.5%, p = 0.182). However, there was no significant difference in body mass index (BMI) between OU and OR, with averages of 22.9 ±  4.5 and 22.5 ±  4.3, respectively (p = 0.001). Similarly, depression scores were comparable between the two groups (OU: 7.05 ±  1.53, OR: 7.00 ±  1.48, p = 0.129).

### Successful aging

The level of successful aging was significantly higher among urban-dwelling older adults (OU) of both sexes compared to their rural counterparts (OR), with scores of 4.32 ±  1.44 versus 3.85 ±  1.24, respectively (p < 0.001). Regardless of location, men were more likely to age successfully than women, with OU men scoring 4.62 ±  1.41 and women 4.03 ±  1.41 (p < 0.001), while for OR, men scored 4.13 ±  1.25 and women 3.62 ±  1.18 (p < 0.001). Stratified analysis by gender revealed heterogeneity in the factors predicting successful aging. Furthermore, S1 Fig illustrates that OR had lower Successful Aging Index (SAI) scores compared to OU across almost all age groups. However, as age increases, the difference between the two groups tends to converge. For both OU and OR, SAI scores tend to decrease with increasing age.

## Discussion

The current study has revealed significant urban-rural disparities in successful aging among the older population in China, with average SAI scores 4.32 ±  1.44 for urban older adults and 3.85 ±  1.24 for rural older adults, respectively (p < 0.001). In summary, there are pronounced differences in successful aging across various dimensions between urban and rural older adults in China. Older adults residing in urban areas outscored those in rural areas on most components of successful aging, leading to higher overall successful aging scores in urban regions for both genders. More specifically, older individuals in urban areas were found to be more physically active (p < 0.001), more adherent to the Mediterranean diet (p < 0.001), less likely to smoke (p = 0.018), more socially engaged (p < 0.001), and possessed higher levels of education (p < 0.001) and financial status (p < 0.001). However, it was observed that older individuals in urban areas had higher obesity rates (p = 0.182), albeit with no significant statistical difference, and a greater number of cardiovascular disease (CVD) risk factors (p < 0.001). Urban and rural older adults had similar depression scores (p = 0.129). Additionally, men tended to age more successfully than women in both settings, suggesting that gender could be a predictive factor for health status in later years, irrespective of the living environment. These findings underscore the need for targeted policies to address the disparities in successful aging between urban and rural areas. This includes the enhancement of social security systems in both urban and rural regions, adjustments to population policies, and the promotion of harmonious urban-rural economic and social development.

Our research findings indicate that the older adults in urban areas are aging more successfully than those in rural areas. A plausible explanation for this disparity could be the relatively slower economic development in rural regions, which has resulted in a severe shortage of resources such as sports facilities, pocket parks, community venues, and hospitals. Furthermore, the economic constraints in rural areas impose greater restrictions on the work opportunities for older adults, leading to a lower participation rate in excursions and organized activities among the rural older adults. As demonstrated in our study, urban-dwelling older individuals are more physically active, a finding that aligns with existing literature on physical activity among urban and rural residents in China [24–26]. Physical activity is not only a crucial promoter of health and a fundamental component of a healthy lifestyle but also an effective avenue for social participation and interaction. Numerous studies have highlighted the benefits of physical activity (PA) on various aspects of well-being among older adults, including subjective well-being [27], life satisfaction [28], health-related quality of life [29,30], health literacy [31], cognitive status [32–34] and mental health [35]. Therefore, physical activity and social engagement are vital for promoting successful aging, and it is imperative for the government to create conducive conditions by providing accessible sports facilities, venues, and a supportive social environment for older adults.

In addition to the disparities in physical activity and social engagement, economic status and lifestyle appear to be contributing factors to the differences in successful aging between urban and rural older populations. Our study reveals that urban older adults enjoy a more favorable economic status (23.9% vs. 16.8%, p < 0.001), which enables them to participate in annual excursions, engage in organized activities more frequently, and consume nutritious food more regularly in their daily diets. Moreover, urban older individuals exhibit higher health literacy compared to their rural counterparts [36]. This heightened awareness likely influences their dietary choices, as OU’s dietary habits more closely resemble the Mediterranean diet, which is known for its health benefits. This is further supported by a similar study indicating that urban residents consume less salt and have a higher intake of vegetables and fruits than rural residents. Additionally, urban residents’ consumption of meat, oil, and alcohol is significantly lower than that of rural residents, with both differences being statistically significant (P < 0.001) [37]. These findings underscore the importance of economic stability and health literacy in shaping dietary habits and overall health outcomes among older adults. The government and relevant authorities should consider these factors when developing policies aimed at promoting healthy aging and improving the quality of life for all older adults, regardless of their place of residence.

Men, regardless of their location, are aging more successfully than women, with average scores of 4.62 ± 1.41 for urban men, 4.03 ± 1.41 for urban women, 4.13 ± 1.25 for rural men, and 3.62 ± 1.18 for rural women (all p < 0.001), indicating that successful aging is characterized by gender heterogeneity. This finding aligns with the results from other studies in the literature [5,38,39]. A plausible explanation for this disparity could be from socioeconomic status differences, gender roles and expectations differences. Men often have a higher socioeconomic status and have higher pension incomes, which impacts their health conditions. Due to historical and cultural factors, men may occupy a stronger position in the labor market, leading to lower economic rewards for women compared to men, and this economic inequality may translate into health disparities. In addition, traditional gender roles and expectations may impact the successful aging of both men and women. For instance, men may be more involved in outdoor activities and social events, while women may take on more household chores and caregiving responsibilities, and these role assignments could affect their health and well-being. For both urban and rural older populations, there is a tendency for SAI scores to decline as age increases, which is consistent with other research [5,39]. The underlying reasons for this trend may include the gradual deterioration of organ function and the reduction in physiological and biochemical reserve capacity associated with aging. These factors increase the vulnerability to physical and psychological diseases, thereby diminishing the quality of life in old age. Concurrently, older individuals face a growing number of age-related diseases, which can limit their ability to perform daily activities, complicating their journey towards successful aging. These insights suggest that policy-making should prioritize additional attention towards women and older individuals of advanced age. By addressing the specific needs and challenges faced by these groups, policies can be more effective in promoting successful aging and enhancing the overall well-being of the aging population.

Cardiovascular disease (CVD) is indeed a significant health concern with rising prevalence and incidence rates globally, and this trend is also observed in China. According to the Global Burden of Disease Study 2017, CVD is the leading cause of morbidity and mortality, responsible for nearly one-third of all deaths worldwide in 2016. In China, it is estimated that by 2020, the number of CVD patients reached 330 million, and CVD accounted for 48.00% of deaths in rural areas and 45.86% in urban areas [40], highlighting its leading position in disease mortality among both urban and rural residents. Our findings indicate that OU are at a higher risk of developing CVD compared to OR (0.73 ±  0.82 vs. 0.54 ±  0.69, p < 0.001), with significantly higher rates of hypertension (p < 0.001), diabetes (p < 0.001), and dyslipidemia (p < 0.001). This disparity could be attributed to several factors. Firstly, urban areas tend to have higher detection rates due to better access to healthcare facilities and services, which may lead to the identification of more cases. Secondly, urban areas often suffer from more severe air pollution, which is a known risk factor for CVD. Studies based on daily air pollution data and causes of death in 272 Chinese cities from 2013 to 2015 demonstrate that increased exposure to PM 2.5 and coarse particulate matter, as well as elevated concentrations of ozone, sulfur dioxide, nitrogen dioxide, and carbon monoxide, raise the risk of death from CVD, coronary heart disease, and hypertension [41–44]. Additionally, data on hospitalization rates and two-week visit rates for diabetes in China show an upward trend [45], with urban rates exceeding those in rural areas. The China Health and Nutrition Survey (CHNS) data from 1991 to 2015 reveal that the standardized age detection rate of hypertension in Chinese adults aged above 18 increased from 30.1% to 43.1% [46], indicating a growing burden of CVD risk factors. In conclusion, the higher risk of CVD in urban areas, as observed in our study, is multifaceted, involving both lifestyle factors and environmental exposures. It is crucial to address these disparities through targeted public health interventions, improved health literacy, and policies aimed at reducing air pollution and enhancing access to healthcare services, particularly for vulnerable populations.

Overall, the research presented offers valuable insights for the fields of policy-making and public health. It underscores the importance of government efforts to bridge the urban-rural divide and enhance the well-being of older adults. The following recommendations are advised for policymakers. Firstly, the government should actively work towards balanced development between urban and rural areas to ensure equitable access to resources and opportunities. Secondly, efforts should be made to encourage healthier lifestyles among older adults. This includes improving access to facilities that support physical activity, such as pocket parks and community centers. Thirdly, investment in infrastructure that caters to the needs of older adults is crucial. This encompasses the development of community libraries, which can serve as hubs for social interaction and lifelong learning, and the establishment of psychology clinics to address mental health needs. Fourthly, creating environments that foster social activity is essential for the successful aging of older adults. This can be achieved through community programs and services that encourage engagement and reduce isolation. Lastly, addressing environmental factors, such as air pollution, is vital to improving health outcomes. Policies aimed at reducing pollution and promoting green spaces can have a significant impact on the health of urban residents. By implementing these measures, the government can support older adults in maintaining better well-being and facilitate successful aging, regardless of their location. This approach not only enhances the quality of life for individuals but also contributes to the overall health and vitality of the community.

## Strengths and limitations

To our knowledge, this study is the first to provide a multidimensional and comprehensive analysis of successful aging levels among older adults in both urban and rural China from an urban-rural differences perspective and by constructing SAI, it offers a novel, pragmatic and quantitative approach to understanding urban-rural differences in China, given that the multidimensional nature of the SAI, which integrates social, psychological, clinical and lifestyle factors, and offers a reasonable and evidence-based framework for evaluating aging outcomes. We have emphasized this point in the discussion section and highlighted the SAI’s utility in identifying specific areas (e.g., physical activity or dietary patterns) where targeted interventions could promote successful aging. All in all, this pioneering work sets a foundation for future research in this area. The findings of this study could serve as a benchmark for future studies aiming to expand upon the observations made here, offering a valuable starting point for comparative analyses and further investigation.

However, several limitations should also be acknowledged. Firstly, a significant limitation of this study is its cross-sectional design, which precludes the ability to infer causal relationships between the factors examined and the outcomes observed. Longitudinal studies would be better suited to establishing cause-and-effect relationships. Secondly, the measurement of the successful aging index in this study was not exhaustive, as it did not encompass cognitive behavior, mobility, physical function, self-assessed quality of life, and self-rated health of the participants. These factors are often integral to characterizing successful aging and overall health, in addition to the low probability of disease and active engagement with life. Thirdly, the cumulative successful aging index developed in this study, which is based on simply adding the presence of common determinants, may not accurately estimate the successful aging status of individuals. A more nuanced approach to measuring successful aging is needed. Future studies are recommended to better understand the causal relationships between various factors and successful aging outcomes, to expand the scope of the successful aging index to include additional factors such as cognitive behavior, and to employ more sophisticated methodologies for calculating the successful aging index to provide a more accurate representation of an individual’s aging status. By addressing these limitations in future research, we can gain a more comprehensive understanding of successful aging among older adults in China and develop more effective policies and interventions to support this demographic.

## Conclusion

The study reveals significant disparities in successful aging between urban and rural older adults in China, with urban areas demonstrating higher rates of successful aging across various dimensions. This urban-rural divide is further compounded by gender differences, where men exhibit more successful aging outcomes compared to women, regardless of their residential location.

These findings underscore the need for targeted interventions and policy considerations that address the unique challenges faced by rural and female older adults. The disparities highlight the importance of equitable resource allocation, improved access to healthcare, and the promotion of healthy lifestyles to support successful aging for all older adults, irrespective of their gender or place of residence. The conclusion drawn from this study is that while urban older adults in China are aging more successfully, there is a pressing need to bridge the gap between urban and rural areas and between genders to ensure that all older adults have the opportunity to age successfully. This calls for a concerted effort to understand and address the underlying factors contributing to these disparities, with the goal of enhancing the quality of life and well-being of older adults across the board.

## Supporting information

S1 Fig Combined scatter plots with quadratic lines showing the relationship between SAI and age by place of residence. (TIF)
