# Supplementary figures and images for: Urban-rural differences in the successful aging among older adults in China
Source: PLoS One. 2025 Mar 20;20(3):e0319105. doi: 10.1371/journal.pone.0319105 (PMC11925467; doi:10.1371/journal.pone.0319105)

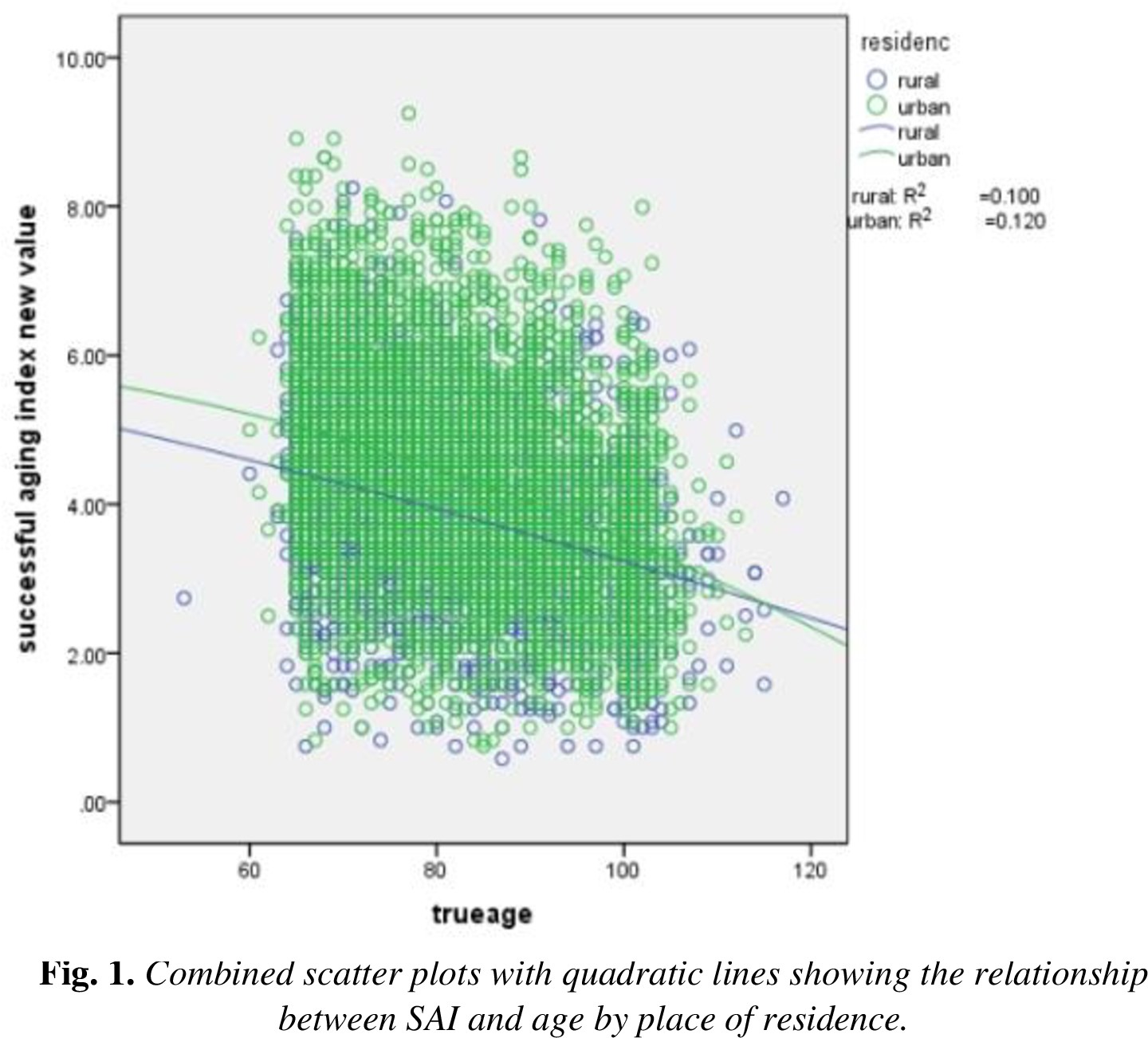

Supplement: S1 Fig — (TIF) [file pone.0319105.s001.tif]
